# Hardware Factors Influencing Strength of Parts Obtained by Fused Filament Fabrication

**DOI:** 10.3390/polym11111870

**Published:** 2019-11-13

**Authors:** Vladimir E. Kuznetsov, Azamat G. Tavitov, Oleg D. Urzhumtsev, Mikhail V. Mikhalin, Alexander I. Moiseev

**Affiliations:** 1 Department of Physical Metallurgy of Non-Ferrous Metals, National University of Science and Technology “MISIS”, Leninskiy Prospekt 4, NUST MISIS, 119049 Moscow, Russia; aztapps@gmail.com (A.G.T.); darikcr@gmail.com (O.D.U.); mih.mikhalin@ya.ru (M.V.M.); 2 Samsung Research Russia, Dvintsev St.12 -1500, 127018 Moscow, Russia; moiseev-ai@ya.ru

**Keywords:** fused filament fabrication, fused deposition modeling, interlayer bonding, direct extruder, Bowden extruder

## Abstract

The current paper investigates the influence of the hardware setup and parameters of a 3D printing process on the resulting sample strength obtained through fused filament fabrication (FFF) technology. Three-point bending was chosen as the strength measure for samples printed with the long side oriented along the Z-axis. A single CAD model was converted into NC-programs through the same slicing software to be run on five different desktop FFF 3D printers with filament of the same brand and color. For all the printers, the same ranges of layer thickness values from 0.1 to 0.3 mm and feed rates from 25 to 75 mm/s were planned to be varied. The first four machines considered in the study were off the shelf devices available on the market, and the fifth was a quick prototype of a desktop machine design based on the analysis of pros and cons of the four machines considered. The results of the study show that the hardware setup of a desktop 3D printer can drastically change the influence of basic technological parameters such as feed rate and layer thickness on the interlayer bonding. This means that many of the conclusions drawn from previous studies connecting the technological parameters of the FFF process with the mechanical performance of parts and samples may only be correct for specific hardware setups.

## 1. Introduction

Among other additive manufacturing technologies used, (polymer) material extrusion remains the most widespread in terms of printed volume (46%) and units sold (75%) [1]. Such a great market share is mostly formed of desktop machines, according to [2,3]. The number of desktop 3D printers based on polymer extrusion rose by a million machines within the last two years. The popularity of machines of such kind has been boosted due to its simplicity and low cost: a part is built by adding threads of molten polymer that is extruded through a hot nozzle. The strength of parts obtained by this method depends largely on bonding between the threads forming adjacent layers. The quality of bonding depends, in turn, on the material properties and various parameters of the material extrusion and deposition process.

Before the rise of the RepRap [4,5,6,7] project, the only company making machines based on the principle of material extrusion was Stratasys. It was founded by Scott Crump, who invented the approach [8,9,10] to printing with molten polymer marketed under the FDM^®^ (fused deposition modeling) trademark. The Stratasys printers and accompanying CAM (computer aided manufacturing) systems (slicers) did not allow the users to vary geometrical and technological parameters beyond predefined limits. For example, Stratasys Dimension Elite allows the layer thickness value to be chosen out of two options—0.176 mm (0.15”) and 0.254 mm (0.2”). Infill density also has two options (high and low), while the shell thickness or any other part geometry parameters, extrusion or environment temperature, and printing speed are not configurable at all. Thus, early research [11,12,13,14,15,16,17,18,19,20,21] on 3D printing with molten polymer parts strength was limited by varying the parameters predefined in Stratasys software. After open source printers and slicers were developed, users obtained full control over the process options. Modern slicers allow for any values to be set including those beyond the physical capabilities of a particular 3D printer, to any parameters including but not limited to the layer thickness, hot end and bed temperatures, linear motion speed, acceleration, and cooling fan speed. There are many recent studies on the influence of different parameters on the printed parts’ strength including extrusion temperature [21,22,23,24,25], layer thickness [26,27,28,29,30,31], printing speed [25,29,32], part orientation [27,29,31,33,34,35], and constitution [27,36,37,38,39,40,41]. However, the findings often do not fit together, or are even in direct contradiction. For example, one of the key parameters of the FFF is the thickness of the layers forming the part, which defines the printed part resolution. The study in [29] claims that for all variants of printing part orientation, part strength increases with an increment in the layer thickness, while [30] directly showed the opposite results.

One of the key reasons for this discrepancy is the absence of a common methodology to assess the strength of FFF printed parts. Most studies have been based on the methods described in the existing standards [42,43] for testing monolithic polymer parts, which were not designed to handle parts with anisotropic features [44]. Various papers have used different printing setups: samples of standardized shape and dimensions have been oriented differently on the bed and were printed with the constitution (the subset of all possible parameters describing shell, infill, and horizontal surfaces) and other parameters (nozzle diameter, temperature) being varied. Another feature of these studies is the broad variety of equipment (namely, 3D printers) used to produce the samples for the tests. The authors have often tried to generalize their findings over the whole FFF technology, which might be premature.

The current study aimed:

- to check if different FFF machines (3D printers) might exhibit different dependency on the process parameters on the same shape of sample strength;
- to map out the influence of technological and hardware factors on the strength of the FFF samples and to sketch up the future research design;
- to design a machine prototype that maximizes the positive influence of hardware factors onto interlayer bonding strength without sacrificing other parameters of print quality.

## 2. Machines, Materials, and Methods

### 2.1. Sample Shape and Dimensions

**Figure 1:**
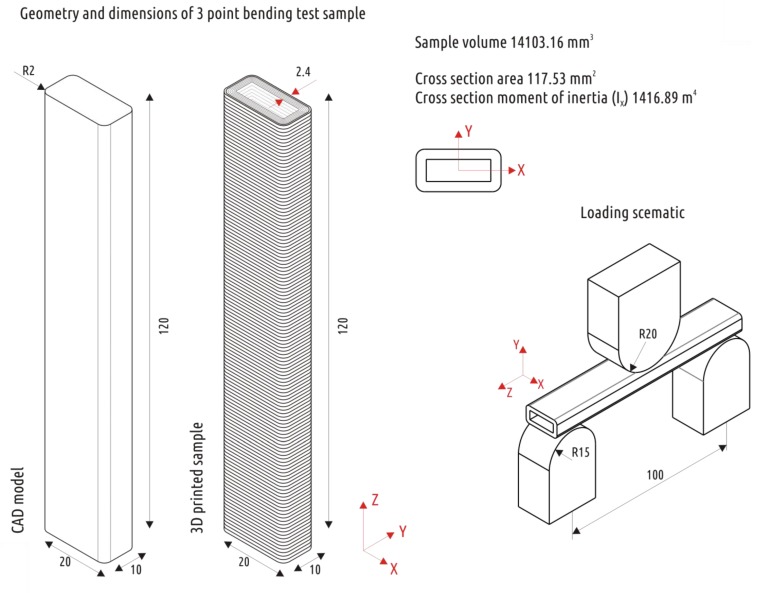
CAD model and 3D printed sample for three point bending.

Recent studies [25,30] have introduced and verified a relatively simple and informative technique for evaluating the interlayer bonding strength of a part obtained with FFF. The technique is based on a three-point bending procedure with loading a sample printed with a long-side oriented along the Z axis (Figure 1). Such an orientation leads to the fact that the maximum stresses in the critical section are acting orthogonally to the layer boundaries, that is, in the direction in which the sample should exhibit the least strength.

The current set of experiments featured tube-shaped samples of rectangular cross-section with 10 mm × 20 mm in size and 120 mm in length with fillets (R = 2 mm) along the long ribs (Figure 1). The tube shape of the sample was provided by setting the “infill” parameter to “0” in the slicer interface program Cura 15.04 (Ultimaker B.V., Geldermalsen, The Netherlands) [45] and turning off the “solid infill top” and “solid infill bottom” checkboxes. The thickness of the tube wall was defined by the “shell thickness” parameter and set to 2.4 mm. The choice of this exact shape and sample size was determined by the desire to obtain data compatible with previous studies [25,30].

### 2.2. Samples Fabrication

#### 2.2.1. Hardware Setup

In the first stage of the study, four different desktop 3D printers were used: Ultimaker 2 (Ultimaker B.V., Geldermalsen, The Netherlands), 3DQ mini (3D Quality JSC, Moscow, Russia), Original Prusa i3 MK3S (Prusa Research s.r.o., Prague, Czech Republic), and Delta WASP 2040 (WASP c/o CSP S.r.l., Massa Lombarda (RA), Italy). The following short names will be used further for convenience: UM2, 3DQ, PRUSA, and WASP for each of the four printers accordingly. All the machines used are based on FFF technology, but there are significant differences in design. The overview is given in Table 1.

In the second stage of the study, the fifth machine was developed based on the analysis of the pros and cons of the commercial printers by analyzing the results of the first stage of study. Since the machine design was based on the observations and conclusions drawn from the first stage of the study, it will be described in the Results and Discussion Section.

#### 2.2.2. Process Parameters

The values of the following 3D printing parameters remained constant during all experiments on all machines:

− nozzle diameter (0.4 mm);
− heated bed temperature (60 °C);
− extrusion temperature (210 °C);
− the first layer thickness (0.3 mm);
− the first layer printing speed (25 mm/s).

The following parameters were varied:

− machine (UM2, 3DQ, PRUSA, and WASP in the first stage, and a custom built device in the second stage);
− printing speed (25, 50, and 75 mm/s);
− layer thickness (in the range between 0.1 and 0.3 mm with a step of 0.05 mm).

The heated bed temperature was not monitored with independent measurements.

The nozzle temperature was checked in the following way. As UM2, PRUSA, and 3DQ use nozzles of the same type with M6 threading, a special nozzle was designed with a built-in K-type thermocouple. A desirable temperature in the range between 100 and 210 °C was set through the printer menu. When the machine was ready, readings from both the printer’s thermal sensor and the thermocouple were recorded. The readings were compared five times for each temperature with a time interval of approximately five minutes between measurements. The following results were obtained (Table 2).

The WASP does not feature an interchangeable nozzle (the whole printhead has to be replaced), thus it was not possible to reproduce the same setup. However, the research on other printers showed that the built-in temperature measurement is accurate enough, at least, in static mode (when the nozzle is heated and no polymer is being extruded).

It is worth mentioning that the sample design (shape and dimensions) was originally verified for use with the UM2 3D printer. No issues with sample stability were observed while printing with the UM2, neither in the current nor earlier studies [25,30]. However, all other machines in the first stage of study experienced problems with the stability of a part being printed at feed rates greater than 25 mm/s. A quick solution was adding brim (20 lines) to the samples printed with 3DQ, PRUSA, and WASP.

For each observation included in the research, a test lot of five samples was obtained. The results were averaged, and two values are represented for values measured in the tables: the mean and the standard deviation.

### 2.3. Material

A turquoise PLA (polylactic acid) filament with gauges of 1.75 and 2.85 mm was used, produced by the REC company (REC, Moscow, Russia). This specific manufacturer of filament was chosen due to the locally produced material. This filament was subject to a differential scanning calorimetry analysis in [25], its glass transition occurs at 70 °C, and its melting point is 151 °C. All spools of 1.75 mm gauge came from the same batch produced in February 2019, according to the labels. All spools of 2.85 mm filament also came from a single batch, produced, however, in June 2018. All spools were sealed in plastic bags with silica gel and packed into cardboard boxes.

In order to obtain the real diameters of the filament utilized, both 1.75 and 2.85 mm filaments were measured with a digital micrometer. For each gauge, 81 diameter measurements were performed at nine points on the three spools, measured in three different axes with an angle of 120° between them. The average diameter for the 2.85 mm gauge was 2.833 mm with a standard deviation of 0.017 mm. The average diameter for the 1.75 mm gauge was 1.729 mm with a standard deviation of 0.016 mm.

### 2.4. Sample Mass Measurement

All samples were weighed before mechanical testing using digital analytical scales ViBRA LF Series (Shinko Denshi Co. LTD, Tokyo, Japan). For samples printed with brim, the brim was torn out before weighing. Measurement results were rounded to two decimal digits. The extrusion efficiency—calculated to measured mass ratio—was computed on that basis. The calculated mass (17.47 g) can be determined by the estimated sample volume (14.10 cm^3^) multiplied by PLA density (1.24 g/cm^3^).

### 2.5. Mechanical Testing

The samples were tested with a universal 50 kN electromechanical testing machine IR 5057-50 (OOO Tochpribor, Ivanovo, Russia) with a test rig for three-point bending. The samples rested on two cylindrical supports of 30 mm diameter with a distance of 100 mm between the centers, and the load was put in the middle of the sample between the supports by a cylinder 40 mm in diameter.

The tests were carried out with the crosshead of the tensile tester moving at a speed of 10 mm/min. During the test, the displacement and load were recorded, the initial load for sample stabilization being set at 5 N. The key parameter registered was the maximum load measured before the samples’ failed (UFL).

For a more general discussion of the results obtained, the normal stresses in the samples at the moment of failure (UFS) were calculated. The stresses were calculated by dividing the maximum moment (*M*) by the section modulus (*W*):

$$UFS=\frac{M}{W},\;\mathrm{MPa}.$$

The maximum moment for testing samples at a distance between the supports (*l*) of 100 mm is

$$M=\frac{F\times l}{4}=0.025F,\;N\cdot m,$$

where *F* is the load in N at the moment of failure (UFL).

The section moment is calculated by using formula:

$$W=\frac{2I}{h},\;m^{3},$$

where *I* is the second moment of the cross-section, calculated with the built-in app of SolidWorks as 1416.89 mm^4^.

Thus, the maximum normal stress in the sample can be calculated from the load by

*UFS* = 0.088*F*,

where *F* is given in N and UFS is in MPa. Since the only method of measuring the mechanical performance of FFF samples was applied, for the sake of simplicity, the term “strength” will be used instead of ultimate flexural strength.

### 2.6. Printing Sample Temperature Evaluation

Temperature distribution over the surface of one sample from each test lot was recorded during the printing process using a FLIR B335 (FLIR Systems, Wilsonville, OR, USA) thermal imager with a resolution of 320 × 240 pixels. The camera was located in front of the printer. Thermal imaging was carried out at the height of the sample being printed between 55 and 65 mm (approximately in the area in which further destruction at the three-point bend occurred). The survey was carried out from a distance of about 30 cm, which is equal to the minimal focal distance documented for the imager.

**Figure 2:**
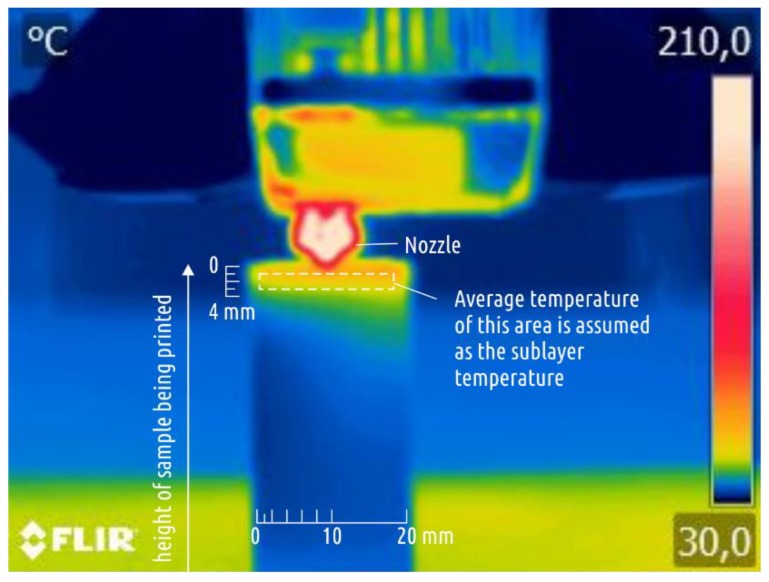
Typical IR image and the area for averaging the temperature with FLIR Tools for use as the sublayer temperature.

The software “FLIR Tools” [46] was used to determine the average temperature on the surface of the sample at a distance of 1 to 3 mm from the lower cut of the nozzle (Figure 2). This temperature was taken as the sublayer temperature.

### 2.7. Macro Imaging of Fractured Surfaces

Images of the fracture surfaces of tested samples were taken with a Sony A6000 (Sony Corp., Tokyo, Japan) digital camera, equipped with Sony E 35 mm f1.8 lens and three macro rings with an overall length of 36 mm. Images were taken with an f/18 aperture, 10 to 15 s exposure, and ISO 100 sensitivity. Displaying the images on a high definition computer monitor resulted in approximately 32× magnification.

## 3. Results and Discussion

### 3.1. Comparison of Commercial Printers

**Figure 3:**
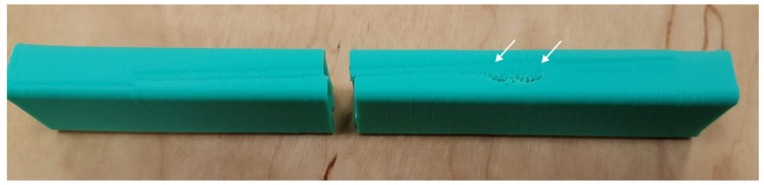
An example of the defects observed on samples fabricated with PRUSA with 0.3 mm layers at a 75 mm/s feed rate.

The full testing program of the current research stage was only completed on the WASP machine and, with certain limitations, on PRUSA. The samples printed on PRUSA with 0.3 mm layers at 75 mm/s linear motion speed had distinctive defects caused by sample oscillation (Figure 3).

**Figure 4:**
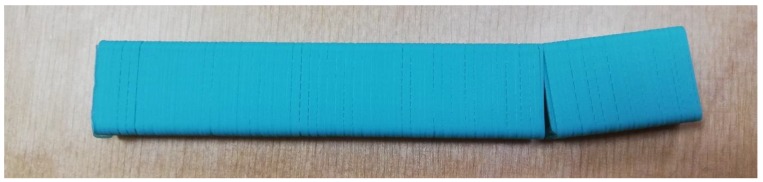
Defects caused by underextrusion on the 3DQ printer with a 0.2 mm layer thickness at a 50 mm/s feed rate.

The defective zone did not lie in the destruction area in the three-point bending test, and thus these samples were not excluded from the scope. Samples printed on the UM2 machine with a speed of 50 mm/s with a layer thickness of 0.25 mm and above, and samples printed with 75 mm/s speed and 0.2 mm layer and above contained explicit underextrusion defects and were excluded. The 3DQ machine could not provide reliable motion at 75 mm/s. Samples printed at 50 mm/s speed suffered from underextrusion (Figure 4) even with a 0.2 mm layer, and thus were excluded. The results obtained are summarized in Table 3, Table 4, Table 5 and Table 6.

**Figure 5:**
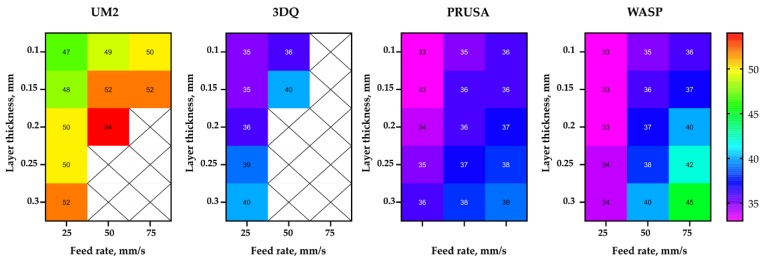
Sublayer temperature (°C) depending on the feed rate, layer thickness, and printer type.

The sublayer temperature of the printed samples differed significantly depending on machine (see Figure 5). This can be explained by the design differences between the machines considered, primarily by the case type and cooling system design and performance.

**Figure 6:**
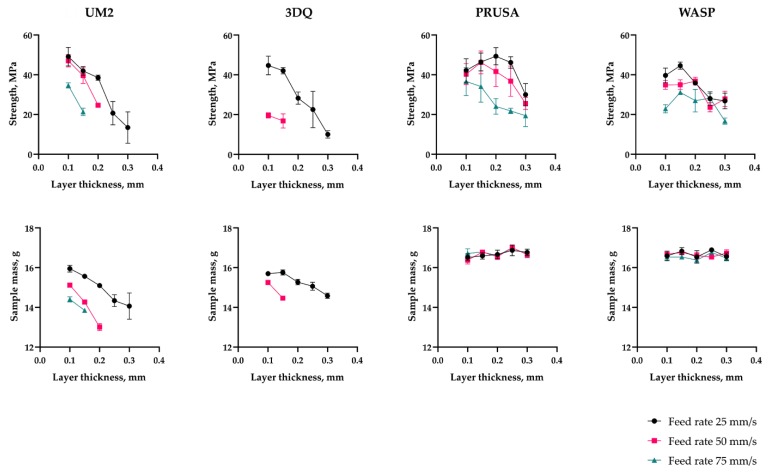
Sample strength and mass depending on layer thickness, feed rate, and printer type.

The maximum strength values recorded for each machine considered were nearly at the same level of ~47 ± 2.5 MPa. However, the dependency of the sample mass and strength on layer thickness and printing speed had different natures on machines of different kinds (see Figure 6).

**Figure A1:**
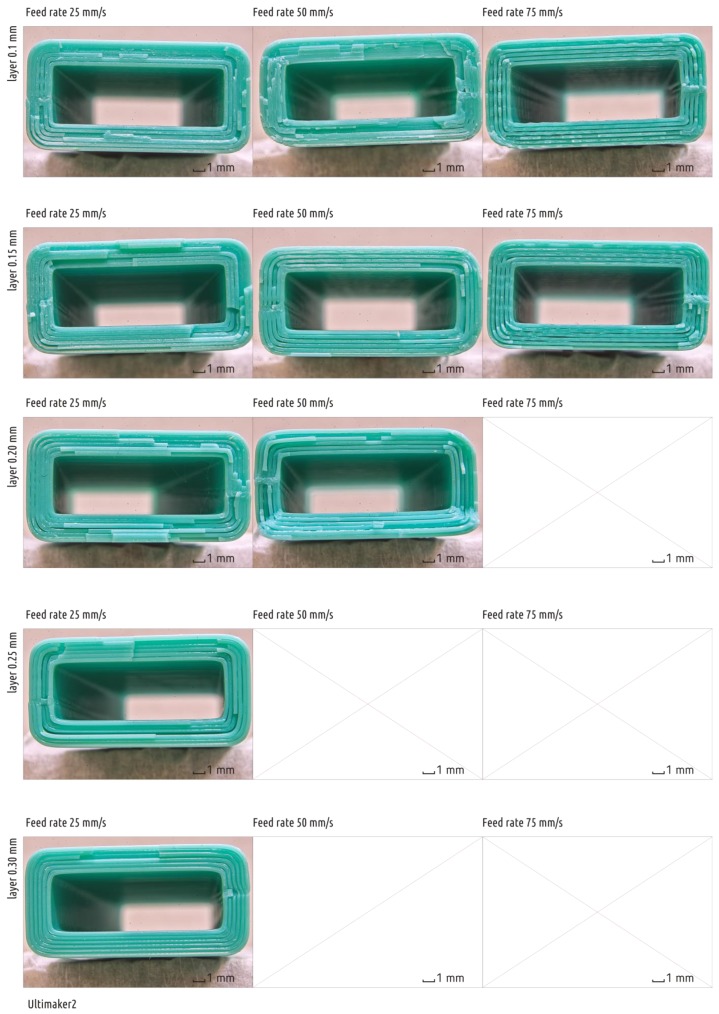
Macro Images of Fracture Surface of Samples from the UM2 Printer.

**Figure A2:**
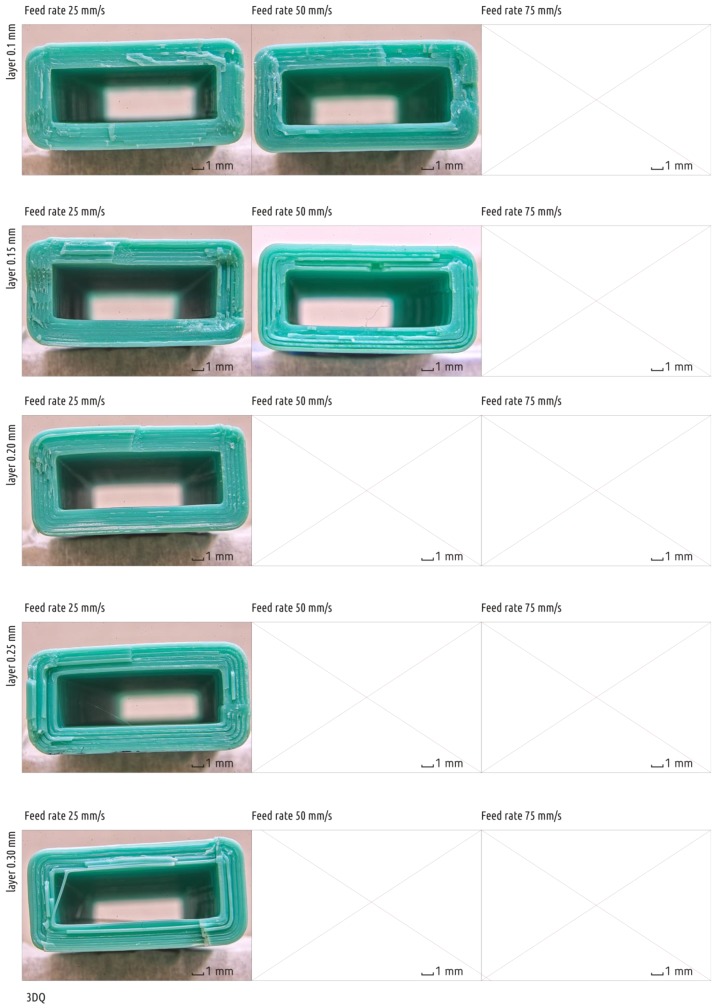
Macro Images of Fracture Surface of Samples from the 3DQ Printer.

**Figure A3:**
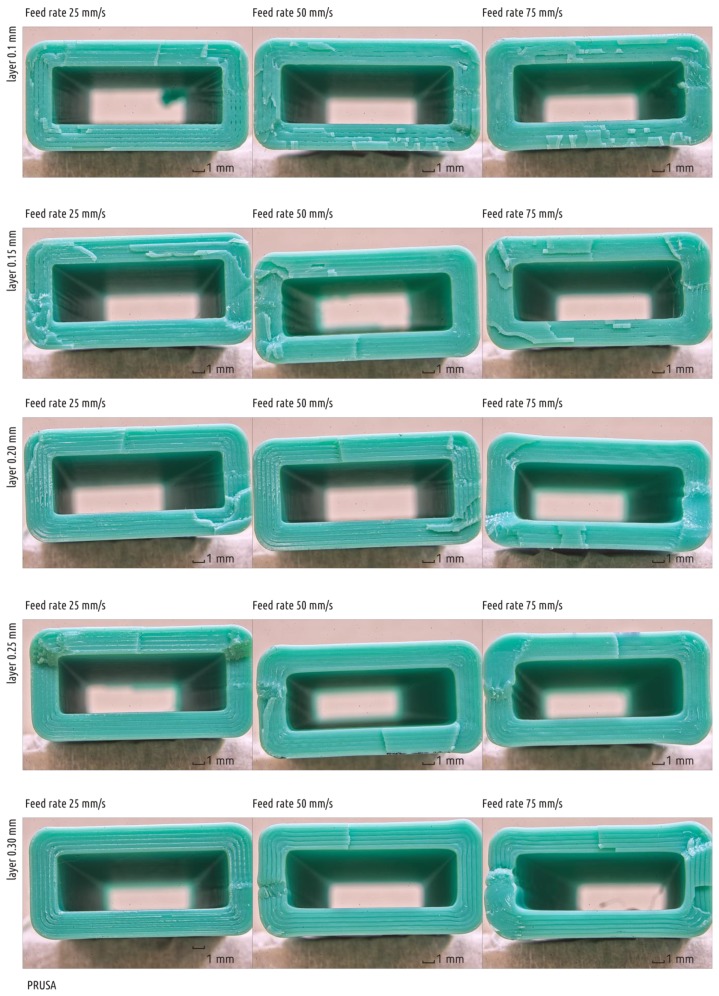
Macro Images of Fracture Surface of Samples from the PRUSA Printer.

**Figure A4:**
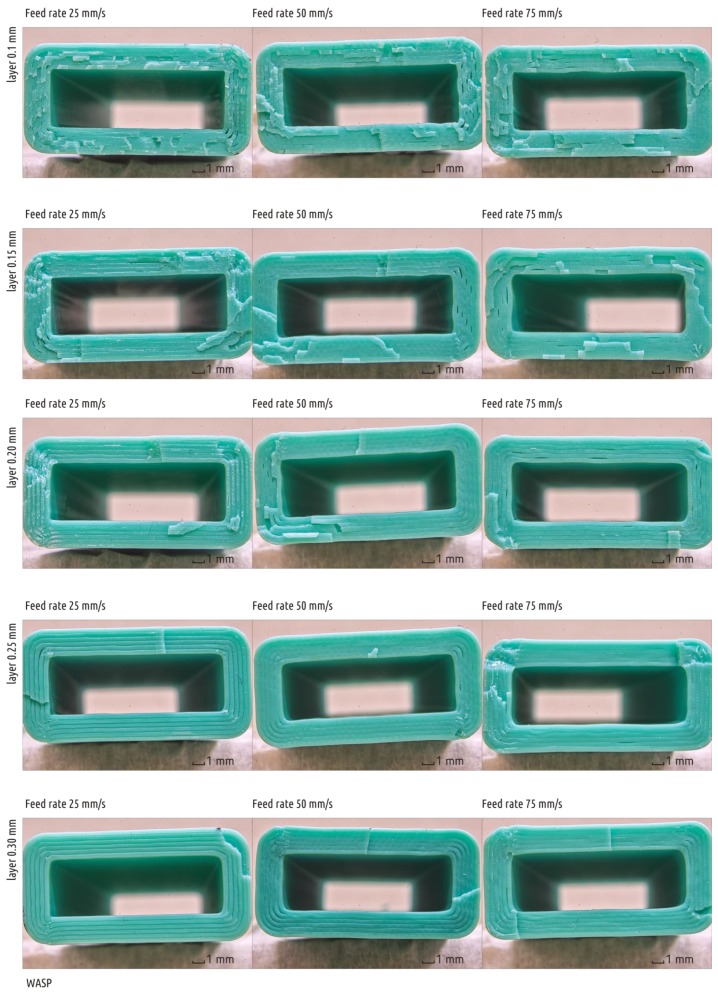
Macro Images of Fracture Surface of Samples from the WASP Printer.

Macro images of the fracture surfaces of samples printed with all four 3D printers are combined in figures shown in Appendix A Figure A1 (UM2), Figure A2 (3DQ), Figure A3 (PRUSA), and Figure A4 (WASP).

#### 3.1.1. Bowden vs. Direct: Extruder Type Influence

**Figure 7:**
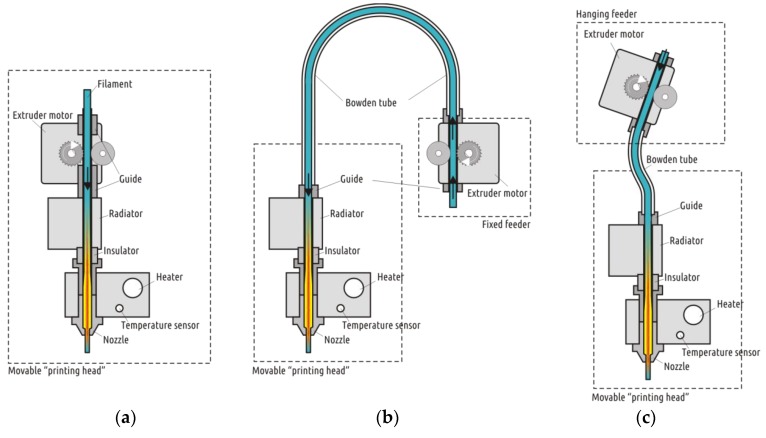
Three types of extruder were considered in the study: direct (**a**), Bowden (**b**), and Bowden with short tube and suspended feeder (**c**).

In the context of the study, type of the extruder (Figure 7) appeared to be the most significant feature of the hardware setup.

**Figure 8:**
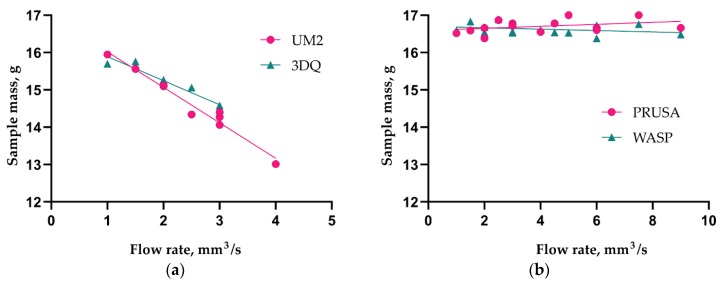
Sample mass dependence on flow rate for printers with Bowden (**a**) and direct or Bowden with short tube (**b**) type of extruder.

As can be seen from the diagram (Figure 8), the extrusion efficiency for extruders of the Bowden type strictly depends on the extrusion resistance. Within a single hardware setup with the Bowden extruder, the extrusion efficiency linearly dropped when increasing the flow rate (with increasing layer thickness or feed rate). The flow rate was calculated by the slicer used in the study (Cura 15.04) by multiplying the layer height, nozzle diameter, and linear motion speed (feed rate). Eventually, increasing flow rate will result in visible underextrusion defects. In contrast, extruders of the direct type work literally in binary mode. When the flow rate is within the hardware productivity limit, it does not affect the extrusion efficiency. When the flow rate goes beyond the limit, the extrusion is interrupted (the extruder motor begins to skip steps or the toothed drive wheel starts to carve filament instead of pushing it forward).

The Bowden extruder with a short tube (WASP) demonstrates similar behavior to the direct extruder (PRUSA); it does not suffer from extrusion resistance. Across the whole range of the flow rate values tested, the mass of samples remained statistically constant. The difference between the Bowden and direct or short tube Bowden extruders is well illustrated by plots of sample mass vs. flow rate (Figure 8). The extrusion efficiency directly affects the contact area between two adjacent layers and thus the bonding strength; this has been verified by the current and previous studies [25,30].

#### 3.1.2. Cartesian vs. Delta: Influence of Motion Scheme

The current study did not reveal either significant advantages or drawbacks of both the Cartesian and delta motion systems. Delta printers are more sensitive to increased printhead weight, thus they often feature Bowden extruders, which can be treated as a drawback. However, the Bowden extruder with shorter tube used on the WASP machine demonstrated results close to the direct extruder on PRUSA.

Considering printing stability and sample surface quality, the following can be said. The coordinate system has a smaller influence on the print quality than the implementation accuracy, especially at higher printing speeds (above 30–40 mm/s). The PRUSA with the Cartesian coordinate system uses the bed moving along the Y axis, resulting in significant vibrations and oscillations of a sample having a high height to bed surface projection ratio while printing at a relatively high speed. This leads to affluxes and other imperfections on the surface, and even grave defects may occur. The delta-shaped WASP printer also has significant vibrations at 75 mm/s speed, thus careful bed setup was required to ensure that the printing sample was not torn away during printing. It also seems that high-speed printing on the WASP machine is not only limited by the mechanics, but by the limited performance of an 8-bit controller that recalculates linear motion in Cartesian coordinates into gentle movement of all three motors. The 3DQ printer with a similar mechanical scheme features a more powerful 32-bit controller, however, high linear feed rates were spoiled by poor linear guides and carriages with loose coupling as well as an arguable hot end holder that allows the nozzle to oscillate. Finally, the Cartesian UM2 contains a bed that is moved along the Z axis, while the lightweight printing head has a similar motion mechanism along both the X and Y axes. There were no stability issues even at high printing speeds. However, high-speed printing (75 mm/s) was limited to layers of 0.1 and 0.15 mm, only due to limited extruder performance.

#### 3.1.3. Thick vs. Thin: Filament Diameter Influence

There was no significant difference discovered for filaments of two popular gauges. The only machine using 2.85 mm filament, the UM2, exhibited results very close to 3DQ. The latter also features a Bowden-type extruder, but consumes the 1.75 mm filament. Further research is needed to set up an experiment involving two similar machines with the only difference being the consumable filament gauge.

#### 3.1.4. Open vs. Closed: Printer Enclosure and Cooling Conditions

Even with the same temperature values set for the nozzles and heated beds and maximum cooling fan speed, there was some variance in the sublayer temperature registered, which has a very high influence on the layers’ cohesion strength [25].

Depending on layer thickness and printing speed, the sublayer temperature varied between 48 to 54 °C on the UM2 machine, with the printing area enclosed from four sides. Printers with an open setup featured lower sublayer temperatures: 34 to 39 °C on PRUSA and 35 to 40 °C on the 3DQ machine. This difference could be explained by a slightly more efficient cooling subsystem in PRUSA.

It can be stipulated that the partially enclosed UM2 machine has certain advantages over open ones (e.g., PRUSA). Therefore, the maximum strength value obtained on UM2 was almost equal to the maximum value for PRUSA. While PRUSA had significantly better extrusion efficiency, perhaps, the higher sublayer temperature compensated for the lack of extruded material.

Finally, the fully enclosed WASP machine exhibited sublayer temperatures in the range of 35 to 45 °C, which is more typical for open type printers. This could be explained by the design and power of the cooling system. Unlike all other machines considered, there is a single cooling fan that pumps the air through the hot end radiator onto the printed part on two sides of the nozzle. With a power consumption of 0.8 A at 12 V, at full speed, it seems that the fan in WASP provides the most intensive airflow of all the machines considered.

### 3.2. Building and Testing a New Machine

One of the conclusions drawn from the first stage results is that none of the machines considered is perfect. In order to approach the ideal, a custom-made machine was created that would only include the strong sides and avoid the weak ones. As a platform, an existing Ultimaker 2 printer was sacrificed (as opposed to building the perfect machine from scratch). As pointed out above, an original UM2 featured the best motion accuracy and fluency. Its only (but significant) problem is the decrease in the extrusion efficiency with increased flow rate values. Replacing the original Bowden extruder with a direct one will obviously dramatically increase the effector (printhead) weight, which in turn is in conflict with the main advantage of the platform, the quick, accurate, and fluent movement. It was thus decided to design a suspended extruder similar to the one found in the WASP printer.

It should be noted that the suspended extruder with a relatively short Bowden tube is not an exclusive feature of WASP 3D printers. The first occurrence of a similar solution named the “flying extruder mod” was found at the RepRap forum as a record made by a user with the *ähM_Key* nickname [47]. The author aimed to extend the speed limitations of FFF technology by using a lightweight effector together with a short guiding tube. A well-known negative feature of Bowden-type extruders is the need to apply relatively long retractions to avoid molten filament dripping from the nozzle during the moves. There are several implementations of a flying extruder used on delta printers; however, we have not found any open source applications of such a scheme on printers with Cartesian geometry.

**Figure 9:**
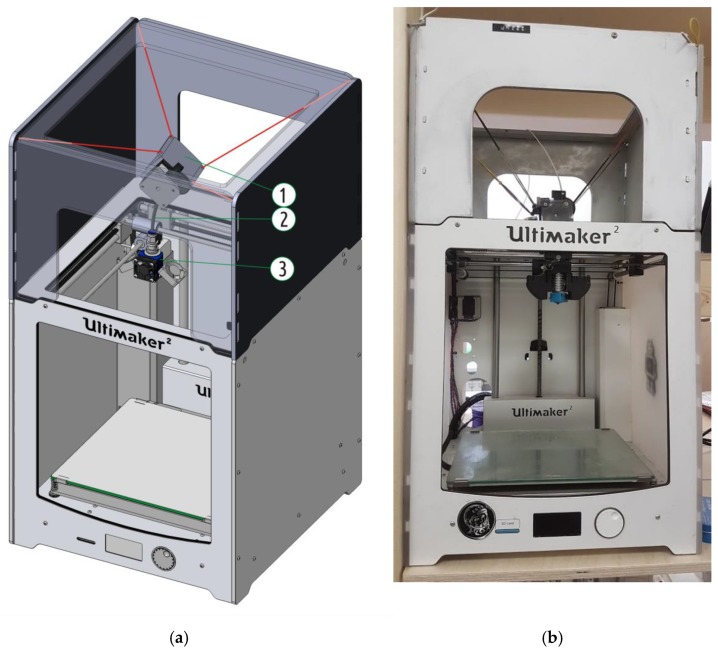
The 3D model (**a**) and the photo (**b**) of the specially designed machine based on a UM2 chassis (front and top parts of the case extension is shown semitransparent): (1) Suspended feeder; (2) short guiding (Bowden) tube; (3) hot end and its mount.

The hot end for the new machine was E3D V6 1.75 mm [48] (E3D-Online Ltd., Chalgrove, Oxfordshire, UK); the same model used on PRUSA and 3DQ printers considered in the current paper. It was mounted on UM2 shafts using an open-source part [49], printed on PRUSA with black PET-G filament (REC company, Moscow, Russia). The cooling fans (one for the hot end radiator and two for the printed part) of the new hot end assembly were taken from the original UM2 machine. The filament supply mechanism was a Titan Extruder (E3D-Online Ltd., Chalgrove, Oxfordshire, UK) [50] equipped with 1.75 mm Bowden adaptor. The mechanism was suspended over the hot end effector on a specially designed frame [51] and four rubber strings (Figure 9), similar to the three-string suspension in WASP. UM2 firmware was not altered, but the E-step parameter was set in accordance with the installation manual [52] from the feeder supplier to 837 steps/mm. Flow parameter for PLA filament was set to 95% in the material setup menu in order to achieve extrusion efficiency (in fact, sample weight) similar to figures on the WASP and PRUSA machines. The hot end design allowed to check the adequacy of the built-in temperature sensor with the same nozzle with the thermocouple, as performed earlier for UM2, PRUSA, and 3DQ. The results are shown in Table 7; the difference between the readings of the built-in temperature sensor and thermocouple is insignificant and can be ignored.

**Figure 10:**
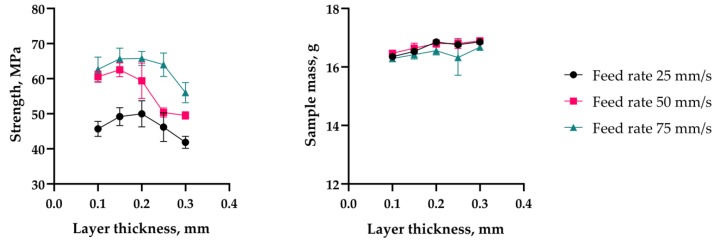
Sample strength and mass depending on layer thickness and feed rate for the UM2SE printer.

The new printer can be named as the Ultimaker 2 with suspended 1.75 mm extruder, or UM2SE in short. This new printer was used to obtain a new set of samples that was later tested following the same routine as described in the first chapter of the research. There were no issues with sample stability or any visual defects occurring across the whole range of layer thickness and printing speed values used. The test results are presented in Table 8 and plotted in Figure 10.

**Figure 11:**
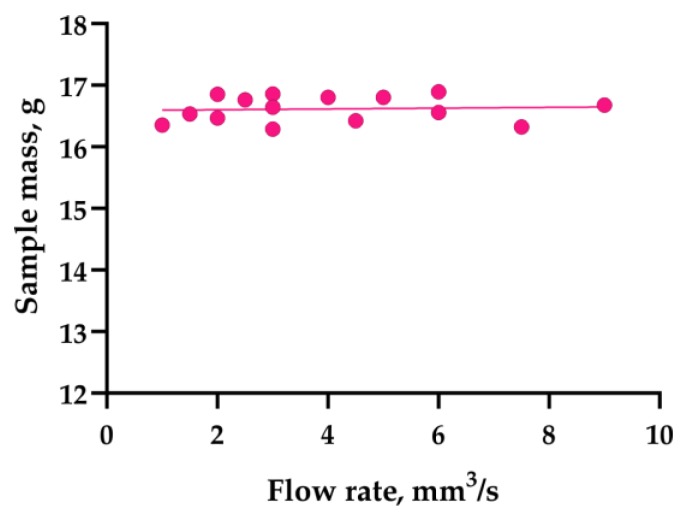
Sample mass dependence on flow rate for the UM2SE printer.

**Figure 12:**
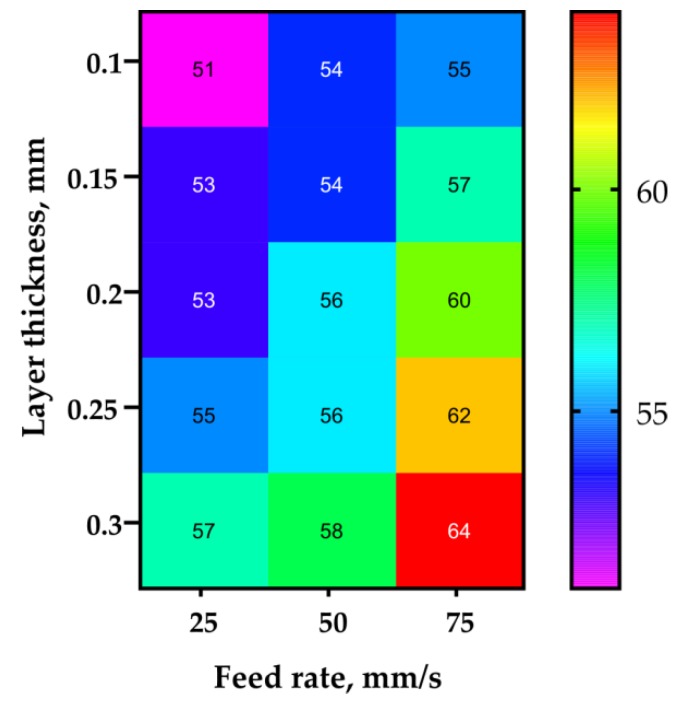
Sublayer temperature (°C) depending on the feed rate and layer thickness for the UM2SE printer.

The maximum average sample strength value obtained with the new printer reached 65.8 MPa. This significantly exceeded the values for the samples from the first stage, which were obtained with the commercial printers. As it can be seen in the chart (Figure 11), the new printer did not suffer from extrusion resistance, and the samples’ mass remained constant regardless the flow rate setting. The new printer also exhibited noticeable increase in the sublayer temperature (Figure 12) when compared with the stock UM2. This effect took place due to the better extrusion efficiency and changed cooling conditions: the suspension system increased the printer case height and might have contributed to better heat retention.

**Figure A5:**
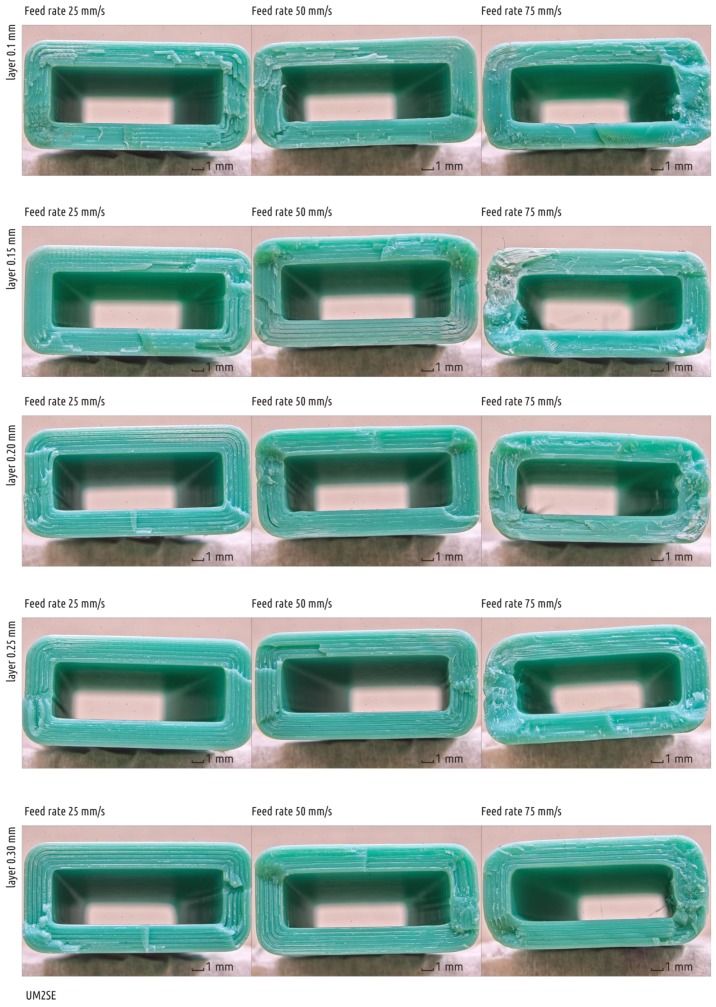
Macro Images of Fracture Surface of Samples from the UM2SE Printer.

There were no stability issues detected, and the correlation between printing speed and sample strength was different than for the other printers previously considered. While PRUSA and WASP make weaker parts as the motion speed increased, UM2SE exhibited the opposite: in the range of speeds chosen, the faster the part was printed, the higher the strength. This phenomenon can be explained by the reduced interval between printing the individual layers and, thus, increased sublayer temperature (Figure 12). Macro images of the fracture surfaces of samples obtained with the UM2SE are shown in Figure A5.

### 3.3. Influence of Technological Parameters with Respect to Hardware Setup

#### 3.3.1. Influence of Printing Speed

In general, increasing the printing speed resulted in a decrease in sample strength for all machines considered in the first stage of the study. Multiple reasons behind this effect might be the cause. There was a single hardware setup (UM2 with 0.6 mm nozzle) considered in a recent study [25] where it was shown that printing speed had a dual effect on bonding strength. On one hand, reducing the layer printing time results in a higher sublayer temperature, which has a positive effect on sample strength. On the other hand, extrusion efficiency is reduced, which adversely affects the strength. The current study resulted in a different situation. First, printing with 0.4 mm nozzles did not result in significant sublayer temperature variance, thus, the positive effect of increasing printing speed was close to zero. Second, a decrease in extrusion efficiency along with increased speed was registered only for Bowden-type extruders. Finally, increasing the printing speed may cause issues with 3D printer motion accuracy. Only the UM2 and the UM2SE machines had no issues with positioning accuracy with increased printing speed. All other machines had one or another issue, although they were different depending on the mechanical design (sample oscillations on a moving heated bed on PRUSA; nozzle colliding into the sample on delta printers, i.e., WASP and 3DQ). These have a negative effect on sample strength.

**Figure 13:**
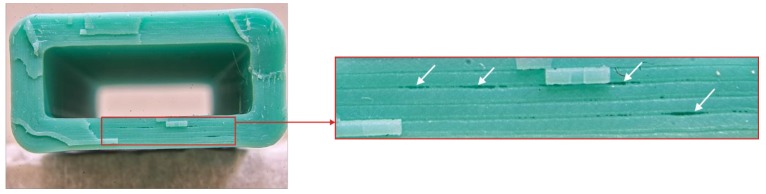
Typical defects visible on the fracture surface of a sample fabricated with PRUSA at 75 mm/s feed rate.

The macroscopic photographs (Figure A1, Figure A2, Figure A3, Figure A4 and Figure A5) of the fracture surface may explain the influence of printing speed on sample strength. Samples obtained on PRUSA with the same layer height with linear motion speeds of 25 mm/s and 50 mm/s exhibited similar properties as well as images of the breakage (see Figure A3). At the same time, the fracture surface of the samples printed at 75 mm/s had specific defects of intermittent voids between the polymer threads. These might be caused by sample oscillation during the process. A section of the thread seems to have shifted along the Y axis (Figure 13), thus forming a void at the boundary with the neighboring thread.

These deviations in thread geometry will adversely affect the sample strength, which was observed in the current study where increasing the printing speed to 75 mm/s on PRUSA significantly reduced the sample strength.

**Figure 14:**
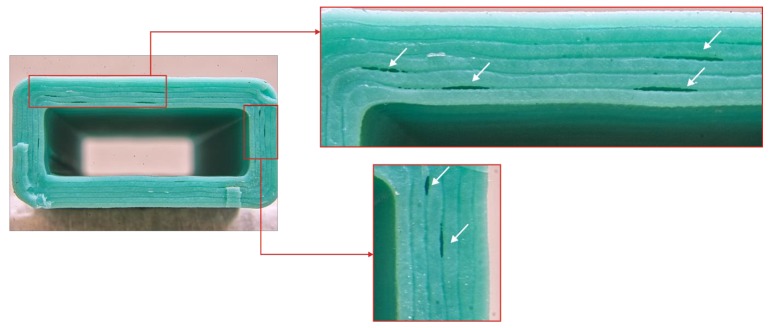
Typical defects visible on the fracture surface of a sample fabricated with WASP at 75 mm/s feed rate.

The WASP exhibited noticeable strength reduction when printing speed was raised from 25 to 50 mm/s, and the difference reached 50% at 75 mm/s. The analysis of zoomed images uncovered printing defects similar to those seen on PRUSA even at 50 mm/s, and further speed increases made them even graver. Unlike PRUSA, the thread was skewed along its full length, which was mostly noticeable on straight chunks (see Figure 14).

These defects might be caused by the hardware specificity of the WASP printer. It is controlled by an 8-bit controller that might not have enough performance to calculate the motions of a delta printing system. While enough accuracy was achieved with a 25 mm/s linear movement rate, higher motion speeds incur a higher computational cost that is not available, and the planner strategy might sacrifice accuracy for overall speed. Thus, the calculations are performed with reduced accuracy and the movement is jittered.

Finally, the samples obtained on the custom designed machine (UM2SE) exhibited distorted and smoothed edges on sample breakage surfaces (Figure A5), but the polymer threads remained parallel to each other. It can be concluded that there were no issues with the sample stability added. Thus, increasing linear motion speed in the range considered positively affected the sample strength. The faster the sample is printed, less intervals occur between printing the adjacent layers, so the higher the sublayer temperature becomes, the stronger the sample.

It should be noted that the effect of strengthening the sample is caused not by increased printing speed itself, but by increased sublayer temperature. If multiple parts are printed at the same time, or the layer cross section has a larger area, the effect of increased printing speed will be different.

#### 3.3.2. Layer Thickness Influence with Respect to Hardware Setup

The distinctive feature of printers with a Bowden-type extruder is the noticeable and sharp drop in unit strength when layer thickness is increased, correlated with reduced extrusion efficiency. This phenomenon is well illustrated by SEM scans of cross-sections of broken parts printed with different setups in [30] as well as breakage surface images obtained in the current study. It can be clearly seen that the voids are becoming wider when increasing layer thickness both on UM2 (Appendix A Figure A1) and 3DQ (Figure A2). Thus, the machines with a common long Bowden tube exhibited a decrease in sample strength when the layer thickness was set to higher values. This fact agrees with the previous study devoted to the UM2 machine [30].

The picture, however, is less clear for printers with a direct extruder (PRUSA) and with a short Bowden tube (WASP, UM2SE). These machines maintain a constant extrusion efficiency rate across the whole range of layer thicknesses and feed rates considered. Thus, increasing the layer thickness did not result in increased voids volume, except for the thickest layers of 0.3 mm, which exhibited the lowest strength values. Even with an extrusion efficiency of 1.0, the threads forming the sample will not be ideally rectangular in cross-section (with sides equal to the layer thickness and nozzle diameter, respectively), but still retain the barrel shape, being squeezed between the nozzle and the sublayer. The morphology of the layer boundary is detailed in [53,54]. Thus, the FFF printing process inevitably results in the formation of voids and cavities at the boundaries of individual threads, and the projection of these voids onto the XY plane grows in area when the layer thickness approaches the nozzle diameter. The fracture surface images (Figure A1, Figure A2, Figure A3, Figure A4 and Figure A5) show that increasing the layer thickness from 0.25 to 0.30 mm is accompanied by a significant increase in the voids’ width. Thus, the minimal values of tensile strength for the samples printed with the thickest layers considered are expected for WASP, PRUSA, and UM2SE.

**Figure 15:**
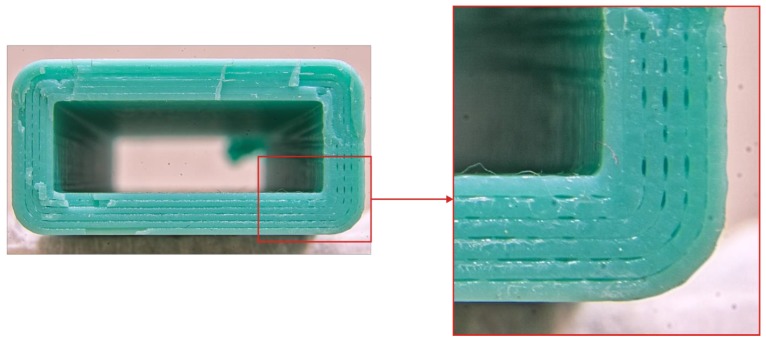
Typical defects visible on the fracture surface of a sample fabricated with PRUSA with thin (0.1 mm) layers.

At the same time, the dependency of the layer thickness in the range between 0.1 and 0.25 mm on sample strength is not clear for PRUSA, WASP, and UM2SE. The difference in the strength of samples printed with 0.15 to 0.25 mm layers on PRUSA is not statistically significant. The fracture surfaces also do not feature visible differences. However, the samples printed with 0.1 mm layers contained specific defect patterns that might be caused by sample oscillation and collisions between the sublayer and the nozzle (Figure 15). With other layer height values, the gap between the nozzle and the sublayer is larger and such collisions do not occur.

A similar situation also took place with the WASP printer. Despite the bed being fixed, oscillations along the Z-axis occurred when the nozzle was moved in the XY plane. Such oscillations become critical when the layer height is low and the nozzle travels close to the sublayer. The fracture images (Figure A4) demonstrated much larger void areas with samples with a 0.1 mm layer height than with other values.

Finally, UM2SE exhibited a statistically insignificant difference in the strength of the samples printed with layer thicknesses between 0.1 and 0.25 mm. The only outlier observed occurred at 50 mm/s with the layer set to 0.25 mm.

For all machines considered that provided a stable extrusion efficiency (PRUSA, WASP, UM2SE), the maximum strength values were obtained with layer thickness of 0.15 or 0.2 mm.

## 4. Conclusions

The mechanical design and build quality of a specific 3D printer can have both a quantitative and qualitative effect on the printed samples’ strength with regard to geometrical and technological parameters of the FFF process. Researchers should be careful when interpreting the results of any experiments: the dependency discovered might only be applicable for the specific class of the FFF devices on which the experiments were run.

Among all the hardware features of the printers considered in the current study, the most important one seems to be the extruder type. There are two kinds of these. The first one provides stable extrusion efficiency across the whole operational range of flow rates: these are direct extruders and Bowden extruders with a short (less than 200 mm) tube. The second type of extruders are those with Bowden type features with a relatively long (over 600 mm) flexible guiding tube. The latter one leads to a decrease in performance (extrusion efficiency) with increased flow rate, which in turn results in a significant reduction in the bonding strength, along with increased layer thickness or feed rate. It might be more correct to stipulate that the distinguishing feature of a FFF 3D printer is the distance between the feeder and the nozzle, rather than extruder type (direct or Bowden).

The second most important hardware feature of a desktop printer is the ability to provide accurate and smooth movement of the nozzle. All machines considered were able to do that with a low printing speed (25 mm/s), but only two of them retained that ability across the whole range of feed rate values, UM2 and UM2SE, using the same chassis.

Analyzing the most prominent advantages and drawbacks of commercial desktop 3D printers tested in the first stage of the study allowed us to design a prototype of a device with a combination of the best properties of the machines considered. The prototype was based on the Ultimaker 2 platform and features an altered hot end and suspended feeder, thus literally combining the strongest features of the machines compared previously. The machine that was designed, built, and tested in the second stage of the study demonstrated noticeably stronger samples when compared to the machines tested during the first stage. Moreover, the new machine demonstrated the opposite effect of the feed rate on the test samples, as opposed to the study of one of the key technological parameters of FFF technology.

## Figures and Tables

**Table 1:** Main parameters of 3D printers in the study.

Machine	UM2	3DQ	PRUSA	WASP
**Scheme**	Cartesian	Delta	Cartesian	Delta
**Filament diameter, mm**	2.85	1.75	1.75	1.75
**Extruder type**	Bowden	Bowden	Direct	Bowden
**Distance from the feeder to the nozzle, mm**	700 mm	750 mm	90 mm	200 mm
**Movement of part being printed**	Along Z	No	Along Y	No
**Power of the heater in the melting block, W**	35	40	40	2*40
**Enclosure**	Front and top open	Fully open	Fully open	Closed
**Bed surface**	Glass	Coated steel + blue scotch tape	Coated steel	Aluminum alloy + blue scotch tape
**Heated bed**	Yes	Yes	Yes	Yes
**Part cooling**	2 × 30 mm fans (12 V 0.1 A)	2 × 40 mm centrifugal fan (12 V 0.13 A)	1 × 50 mm centrifugal fan (5 V 0.25 A)	1 × 40 mm axial (12 V 0.8 A)
**Acceleration settings used**	3000 mm/s^2^ (XYZ)	500 mm/s^2^ (XYZ)	3000 mm/s^2^ (XYZ)	3100 mm/s^2^ (XYZ)
**Control board/firmware**	8 bit ATMega2560 (Marlin-based)	32 bit Smoothieboard compatible	8 bit ATMega2560 (Marlin-based)	8 bit ATMega2560 (Marlin-based)

**Table 2:** Preset and measured temperature at nozzles, the mean and the standard deviation are in parentheses.

Preset temperature, °C	Measured temperature, °C		
	PRUSA	UM2	3DQ
100	102 (2)	99 (2)	97 (3)
150	151 (2)	151 (2)	148 (2)
200	201 (1)	200 (2)	198 (3)
210	211 (2)	211 (2)	208 (2)

**Table 3:** Experimental results for the UM2 3D printer.

Layer Thickness, mm	Feed Rate, mm/s	Sample Mass, g		Extrusion Efficiency	Sublayer Temperature, °C	Strength, MPa	
		Mean	SD			Mean	SD
0.1	25	15.95	0.17	0.91	47	49.1	4.7
0.15	25	15.56	0.07	0.89	48	41.91	2.23
0.2	25	15.10	0.07	0.86	50	38.54	1.25
0.25	25	14.34	0.3	0.82	50	20.7	5.86
0.3	25	14.06	0.66	0.8	52	13.43	7.9
0.1	50	15.12	0.09	0.87	49	47.01	3.12
0.15	50	14.27	0.09	0.82	52	39.56	3.93
0.2	50	13.01	0.18	0.74	54	24.61	0.88
0.1	75	14.4	0.13	0.82	50	34.64	1.32
0.15	75	13.85	0.05	0.79	52	21.3	1.87

**Table 4:** Summary of the experiments for the 3DQ 3D printer.

Layer Thickness, mm	Feed Rate, mm/s	Sample Mass, g		Extrusion Efficiency	Sublayer Temperature, °C	Strength, MPa	
		Mean	SD			Mean	SD
0.1	25	15.70	0.06	0.90	35	44.73	4.67
0.15	25	15.76	0.13	0.90	35	42.06	1.61
0.2	25	15.27	0.14	0.87	36	28.25	3.07
0.25	25	15.06	0.20	0.86	39	22.57	9.12
0.3	25	14.58	0.13	0.83	40	10.08	1.85
0.1	50	15.25	0.1	0.87	36	19.62	1.31
0.15	50	14.46	0.01	0.83	40	16.84	3.56

**Table 5:** Summary of the experiments for the PRUSA 3D printer.

Layer Thickness, mm	Feed Rate, mm/s	Sample Mass, g		Extrusion Efficiency	Sublayer Temperature, °C	Strength, MPa	
		Mean	SD			Mean	SD
0.1	25	16.52	0.17	0.95	33	42.17	5.92
0.15	25	16.59	0.16	0.95	33	46.41	4.53
0.2	25	16.66	0.22	0.95	34	49.32	4.32
0.25	25	16.87	0.27	0.97	35	46.16	2.92
0.3	25	16.77	0.16	0.96	36	29.99	5.61
0.1	50	16.38	0.19	0.94	35	40.3	5.29
0.15	50	16.78	0.07	0.96	36	46.23	5.74
0.2	50	16.55	0.15	0.95	36	41.65	7.59
0.25	50	17.00	0.07	0.97	37	36.75	7.6
0.3	50	16.66	0.16	0.95	38	25.55	3.02
0.1	75	16.72	0.23	0.96	36	36.61	7.09
0.15	75	16.78	0.07	0.96	36	34.06	7.9
0.2	75	16.6	0.1	0.95	37	24.05	3.91
0.25	75	17.0	0.07	0.97	38	21.77	1.34
0.3	75	16.66	0.15	0.95	39	19.39	5.52

**Table 6:** Summary of the experiments for the WASP 3D printer.

Layer Thickness, mm	Feed Rate, mm/s	Sample Mass, g		Extrusion Efficiency	Sublayer Temperature, °C	Strength, MPa	
		Mean	SD			Mean	SD
0.1	25	16.59	0.24	0.95	33	39.67	3.71
0.15	25	16.83	0.18	0.96	33	44.59	1.8
0.2	25	16.54	0.32	0.95	33	35.85	0.48
0.25	25	16.89	0.04	0.97	34	28.03	3.39
0.3	25	16.56	0.18	0.95	34	26.84	3.99
0.1	50	16.68	0.02	0.95	35	34.88	2.19
0.15	50	16.76	0.06	0.96	36	34.99	2.48
0.2	50	16.6	0.17	0.95	37	36.83	1.93
0.25	50	16.53	0.11	0.95	38	23.61	2.25
0.3	50	16.73	0.17	0.96	40	27.81	3.87
0.1	75	16.53	0.19	0.95	36	22.85	2.08
0.15	75	16.54	0.06	0.95	37	31.12	0.82
0.2	75	16.38	0.1	0.94	40	26.96	5.67
0.25	75	16.76	0.08	0.96	42	28.16	2.38
0.3	75	16.48	0.15	0.94	45	16.66	1.6

**Table 7:** Preset and measured temperature at the nozzle of UM2SE.

Preset Temperature, °C	Measured Temperature, °C	
	Mean	SD
100	98	3
150	150	3
200	198	2
210	209	4

**Table 8:** Experiments summary for the UM2SE 3D printer.

Layer Thickness, mm	Feed Rate, mm/s	Sample Mass, g		Extrusion Efficiency	Sublayer Temperature, °C	Strength, MPa	
		Mean	SD			Mean	SD
0.1	25	16.35	0.03	0.93	51	45.67	2.14
0.15	25	16.53	0.01	0.94	53	49.19	2.54
0.2	25	16.85	0.06	0.96	53	49.98	3.72
0.25	25	16.76	0.1	0.96	55	46.18	4.09
0.3	25	16.47	0.08	0.96	57	41.86	1.72
0.1	50	16.64	0.04	0.94	54	60.57	1.6
0.15	50	16.8	0.17	0.95	54	62.54	1.98
0.2	50	16.8	0.13	0.96	56	59.4	5.04
0.25	50	16.89	0.17	0.96	56	50.37	1.32
0.3	50	16.47	0.03	0.97	58	49.51	1.05
0.1	75	16.29	0.05	0.93	55	62.65	3.5
0.15	75	16.42	0.15	0.94	57	65.65	3.05
0.2	75	16.56	0.15	0.95	60	65.79	2.01
0.25	75	16.32	0.61	0.93	62	63.98	3.35
0.3	75	16.68	0.04	0.95	64	56.03	2.88

## Nomenclature

In the current work, the following notions are introduced and the following abbreviations are used:

FFF	Fused filament fabrication is a technology of digital additive manufacturing based on the extrusion and layered deposition of melted thermoplastic. From a technological point of view, *FFF* is the same as *FDM*^®^—fused deposition modeling, the only difference being that while the *FDM*^®^ is a registered trademark and thus applies to Stratasys machines only, *FFF* is a term coined inside the RepRap community;
PLA	Polylactic Acid, a polymer commonly used in FFF process
UFL	Ultimate fracture load, maximum load observed during mechanical testing;
UFS	Ultimate fracture strength, calculated stress in the sample caused by *UFL*;
Sublayer	A layer of plastic already deposited by a *FFF* 3D printer, which acts as a substrate for the layer being deposited at a given moment;
Filament	Plastic in the filament form used as supply in the *FFF* process;
Thread	Extruded and deposited thread of plastic mimicking the 3D printed part;
Feed rate	The linear printing speed, the speed of the nozzle traveling across the XY plane while extruding and depositing the plastic threads;
Flow rate	The volume of plastic delivered through the nozzle per unit of time;
Extrusion efficiency	The ratio of real to calculated mass of 3D printed object;
Underextrusion	A characteristic defect in the *FFF* process resulting in a significant drop in thread thickness or in thread interruptions caused by a shortage of *flow rate* and low extrusion efficiency.
